# The role of buoyancy in the fate of ultra-high-pressure eclogite

**DOI:** 10.1038/s41598-019-56475-y

**Published:** 2019-12-27

**Authors:** Timothy Chapman, Geoffrey L. Clarke, Nathan R. Daczko

**Affiliations:** 1 School of Geosciences, The University of Sydney, Sydney, NSW 2006 Australia; 2 ARC Centre of Excellence for Core to Crust Fluid Systems and GEMOC, Department of Earth and Environmental Sciences, Macquarie University, Sydney, NSW 2109 Australia; 3 Present Address: Earth Science, School of Environmental and Rural Science, University of New England, Armidale, NSW 2351 Australia

**Keywords:** Petrology, Tectonics

## Abstract

Eclogite facies metamorphism of the lithosphere forms dense mineral assemblages at high- (1.6–2.4 GPa) to ultra-high-pressure (>2.4–12 GPa: UHP) conditions that drive slab-pull forces during its subduction to lower mantle conditions. The relative densities of mantle and lithospheric components places theoretical limits for the re-exposure, and peak conditions expected, of subducted lithosphere. Exposed eclogite terranes dominated by rock denser than the upper mantle are problematic, as are interpretations of UHP conditions in buoyant rock types. Their subduction and exposure require processes that overcame predicted buoyancy forces. Phase equilibria modelling indicates that depths of 50–60 km (P = 1.4–1.8 GPa) and 85–160 km (P = 2.6–5 GPa) present thresholds for pull force in end-member oceanic and continental lithosphere, respectively. The point of no-return for subducted silicic crustal rocks is between 160 and 260 km (P = 5.5–9 GPa), limiting the likelihood of stishovite–wadeite–K-hollandite-bearing assemblages being preserved in equilibrated assemblages. The subduction of buoyant continental crust requires its anchoring to denser mafic and ultramafic lithosphere in ratios below 1:3 for the continental crust to reach depths of UHP conditions (85–160 km), and above 2:3 for it to reach extreme depths (>160 km). The buoyant escape of continental crust following its detachment from an anchored situation could carry minor proportions of other rocks that are denser than the upper mantle. However, instances of rocks returned from well-beyond these limits require exceptional exhumation dynamics, plausibly coupled with the effects of incomplete metamorphism to retain less dense low-P phases.

## Introduction

**Figure 1:**
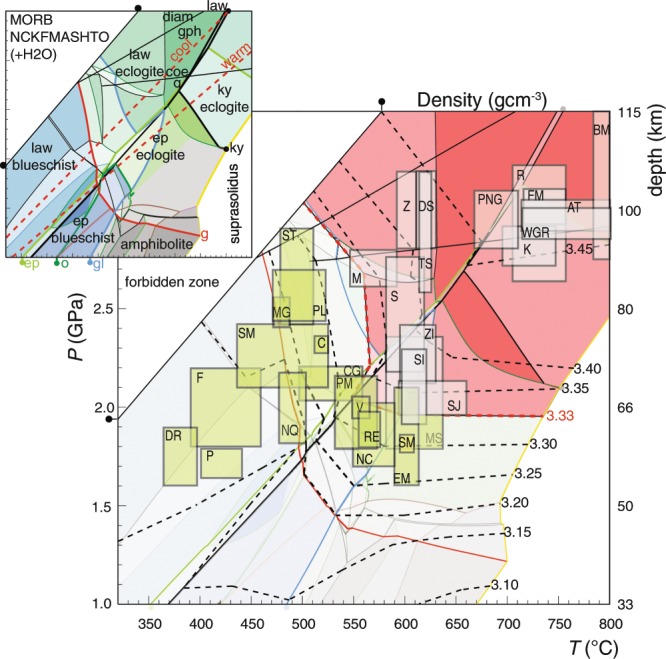
Pressure–temperature (*P–T*) pseudosection for a mid-ocean ridge basalt (MORB), displaying the metamorphic facies for this protolith and the *PT* boundaries of key minerals (inset). Different shading intensity of the fields represents changes in variance of the phase assemblage, the red shading represents fields denser than the ambient mantle. Warm and cool slab-parallel geotherms are displayed for reference (dashed red lines). Isopycnals for the metamorphosed MORB are shown in dashed black lines, with assemblage fields above the density threshold delineated in red (3.33 gcm^−3^). Labelled boxes represent the *P–T* conditions inferred for natural eclogite terranes, which correlate well with the predicted mineral assemblages (Table S2).

The buoyancy of subducted lithosphere is a principle determinant in its descent from, and/or return to Earth’s surface, where less or more buoyant than the enclosing mantle, respectively^1,2^. Rock density varies in relation to progressive mineralogical change during metamorphism, and is a function of the ambient geotherm, depth and rate of subduction. A crucial density threshold is generated by metamorphic changes at eclogite facies conditions (depths ≈ 60–70 km and *P* ≈ 1.8–2.2 GPa), which presents as a point of no-return for mafic crust (Fig. 1)^3,4^. The final destination for most subducted material is shown by seismic tomography to be either the mantle transition zone or the core–mantle boundary^5^. However, the presence of mafic eclogite at Earth’s surface indicates that dense material can return from high-pressure conditions (>1.8 GPa). Most instances of rocks returning from conditions above the quartz–coesite transition (>2.4 GPa), referred to as ultra-high-pressure (UHP), involve predominantly silicic continental lithosphere, which remains positively buoyant to greater depths^2,6^.

The preservation of coesite, diamond and majoritic garnet defining UHP terranes are consistent with their return from depths of 80–160 km (*P* = 2.4–5 GPa)^7–10^. However, interpretations of pseudomorphs after stishovite, exsolved components in garnet, clinopyroxene and olivine or α-PbO_2_-type TiO_2_ with and without majoritic garnet, have led to inferences of burial depths approaching the mantle transition zone (400 km: *P* up to 12–14 GPa) for mixed mafic and felsic terranes^11–18^. The interpreted exhumation of rocks from these depths is problematic in the context of return-limits established through experimentation (~250 km)^19–21^. Such circumstances require the action of an attached and denser “anchor” to firstly reach such conditions, and then another process to restore positive buoyancy and enable their return to Earth’s surface. The proportion of felsic to mafic material in subducted lithosphere is thus crucial to buoyancy relations at various depths, and presents a test for the validity of the UHP record, yet it is seldom quantified^22^. Forward predictions of phase equilibria^23^ for lithosphere comprising varying proportions of lherzolitic mantle and crust of either oceanic or continental composition can establish the pressure–temperature (*PT*) conditions under which the subducted components will become neutrally buoyant in the upper mantle^24–26^. Oceanic crust is represented here by mid-ocean ridge basalt (MORB), and the more diverse continental crust is explored using averaged andesitic and granitic compositions^27^. Crucial preservation thresholds – points of no-return – and their determinative buoyancy agents are established by considering the gross changes involved in differing lithospheric components and validated using detail from the natural record. The predicted preservation thresholds reconcile the range of *PT* conditions commonly recorded by eclogite facies terranes, and enable the veracity of UHP estimates to be queried. The modelled outcomes highlight the importance of identifying robust petrological evidence in buoyant rock types that can be used to corroborate *PT* conditions inferred from elemental exchange equilibria, such as the former stability of wadeite, K-cymrite or K-hollandite, where rocks have been returned from below the predicted preservation thresholds.

## Phase Equilibria Modelling

**Figure 2:**
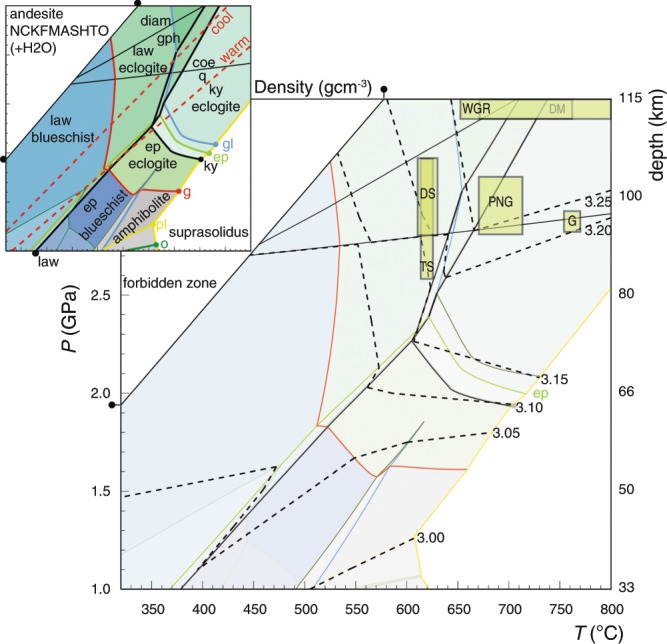
*P–T* pseudosection for a metamorphosed andesite, displaying the metamorphic facies for this protolith and the *PT* boundaries of key minerals (inset). Different shading intensity of the fields represents changes in variance of the phase assemblage. Warm and cool slab-parallel geotherms are displayed for reference (red dashed lines). Isopycnals for the metamorphosed andesite are displayed in dashed black lines with the labelled boxes representing the *P–T* conditions inferred for natural eclogite terranes (Table S2). No red shading is present as all phase assemblages density remain less than the ambient mantle.

**Figure 3:**
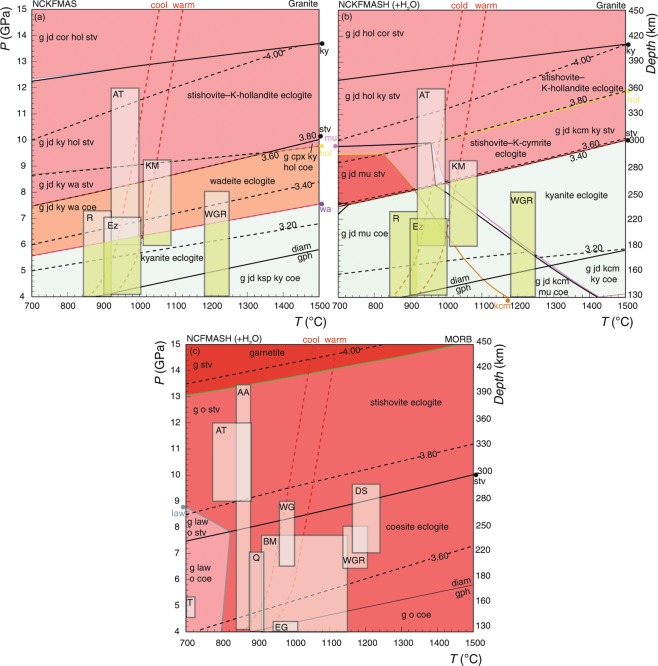
*P–T* pseudosections for metamorphosed dry (**a**) and wet granite (**b**), and MORB (**c**) at upper mantle conditions (*P* = 4–15 GPa). Different shading intensity of the fields represents changes in variance of the phase assemblage. Warm and cool slab-parallel geotherms are displayed for reference (red dashed lines). Isopycnals are displayed in dashed black lines and labelled boxes represent *P–T* conditions inferred for natural UHP terranes (Table S2). Assemblage fields that are denser than the ambient mantle are delineated in red and those with similar density to the ambient mantle in orange.

**Figure 4:**
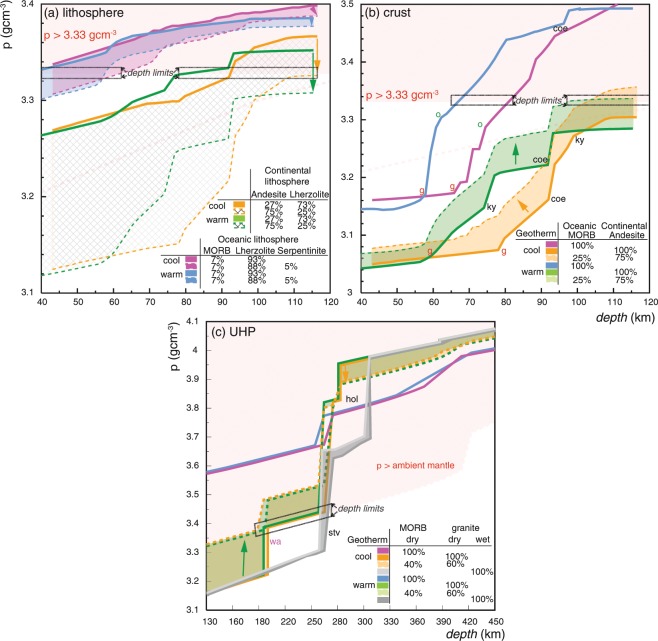
Predicted density of phase equilibria in oceanic and continental lithosphere (**a**) and crust (**b**,**c**) during cool and warm subduction. The effects of differing proportions of crustal components is shown in (**b**,**c**) at various depths. The *PT* boundaries of key minerals are shown for reference, including the lower limit of UHP conditions (quartz–coesite transition). A conjugate diagram illustrating the dependence of density on the lithospheric components for the warm and cool geotherms is shown in Fig. S4.

Pressure–temperature (*P–T*) pseudosections^28,29^ for MORB (Fig. 1), andesite (Fig. 2), granite (Fig. 3) and lherzolite (Fig. S1) represent the metamorphism of oceanic and continental lithosphere during subduction. The simple lithospheric models used here assume 7 km of oceanic and 40 km of continental crust with 93 and 110 km of lithospheric mantle, respectively (Fig. 4). The oceanic scenario represents an example of 100 Myr old subducted lithosphere^30^. Continental crust has more variability in its composition and water content, which can be expected to induce limited departures in its overall density (~0.05 gcm^−3^) compared to that of the average andesite model (see Supplement Fig. S4). Scenarios involving differing proportions of crust (mafic, andesitic and granitic) *and* lithospheric mantle (lherzolite and/or serpentinite) are assessed to establish buoyancy constraints on the lithosphere reaching and returning from extreme depths (Fig. 4).

Ambient upper mantle densities established along an isentropic potential temperature of 1350 °C present a threshold critical to evaluating the buoyancy of subducted lithosphere and its potential return to the surface (Fig. S1). The uniformity of the upper mantle under typical conditions limits variation in its density from 3.33 gcm^−3^ at low-*P* (<2 GPa) equivalent to depths of less than 65 km^3^. However, the density of lherzolite is strongly influenced by the proportion of hydrous minerals (e.g. in serpentinised components); their breakdown is coupled with large changes in density (Fig. S1).

High-pressure conditions induce dense assemblages in crustal rock types (Figs. 1 and 2). The capacity for oceanic crust to exceed the upper mantle density is not dependent solely on it reaching eclogite facies conditions. It is, instead, controlled mostly by high-variance equilibria associated with the breakdown of chlorite and amphibole, and the growth of omphacite and garnet (Fig. 1). These reactions induce densification above 3.33 gcm^−3^ at depths dependent on the ambient geotherm: oceanic crust experiencing warm subduction will be neutrally buoyant at approximately 65 km (2 GPa) *versus* 80 km (2.5 GPa) in scenarios of cool subduction (Fig. 1). Continental crust of andesitic composition can be expected to increase density at a lower rate than MORB crust, as garnet and omphacite modes will be lower (Fig. 2). Steps in the density of such silicic and aluminous material are influenced strongly by kyanite mode and the quartz to coesite transition, with values being less than 3.33 gcm^−3^ across the modelled range (1–4 GPa; Fig. 2).

The calculation of NCKFMASHTO mineral assemblage densities at *P* > 3.5 GPa is limited by our current understanding of activity–composition models at UHP conditions^28,29^. However, the higher-pressure density of felsic and mafic crustal end-members (i.e. granitic and basaltic compositions) can be assessed in the reduced NC(K)FMAS(H) system against that of a lherzolitic upper mantle (Figs. 3 and S1)^29^. Mafic crust can be expected to metamorphose to high-variance, dense equilibria. The stabilisation of stishovite and the dissolution of omphacite into majoritic garnet are predicted to induce densities in basaltic crust that are greater than that of the surrounding lherzolitic mantle (Fig. 3c). Dry granitic crust is predicted to undergo polymorphic transformations of K-feldspar to wadeite at *P* = 5.5–6.5 GPa or depths of 200 to 220 km, and wadeite to K-hollandite near the stishovite transition (280 km: Fig. 3a). For fluid-saturated granitic crust, wadeite is preferentially destabilised by phengite and K-cymrite with or without kyanite depending on temperature (Fig. 3b). The growth of K-hollandite at the expense of K-cymrite occurs at higher-*P* (9–12 GPa) equivalent to burial depths of 280–360 km. The stabilisation of wadeite in dry granitic crust induces assemblage densities close to that of the ambient mantle (3.43 gcm^−3^), that are overall denser than in hydrous assemblages stable at otherwise equivalent conditions (3.20–3.30 gcm^−3^), consistent with previous experimental predictions (Fig. 4c)^3,19–21^.

## Points of No-Return

**Figure 5:**
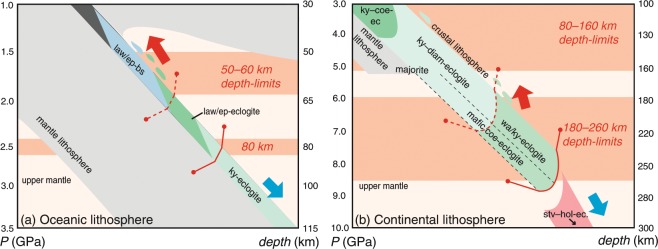
Schematic representation of the predicted depth-limits for the buoyant exhumation of subducted oceanic (**a**) and continental (**b**) crust.

The slab-pull force exerted by subducting lithosphere is mostly controlled by the density of its mantle component^1,4,31^. This is, in turn, principally dependent on the ambient geotherm, as the isopycnals (line of equal density) in lherzolite are controlled by its thermal expansivity (Figs. 3 and S1). Oceanic lithosphere is predicted to be denser than 3.33 gcm^−3^ at much shallower conditions (60 km and *P* = 1.2–1.4 GPa) compared to continental lithosphere (85 km and *P* > 2.6 GPa), on account of higher modes of garnet, omphacite and amphibole in its crustal component (Figs. 1 and 2). At depths below these buoyancy thresholds, much of the lithosphere could be expected to be exhumed or involved in a circumstance of stalled subduction, as the pull-force will be terminated or greatly diminished (Fig. 5)^32–34^. These depth estimates for the points of no return will increase if a reduced proportion of the dense lithospheric mantle is subducted due to tectonic scenarios involving lithospheric attenuation or dismemberment (Fig. 4a)^2,6^.

The majority of exhumed eclogite terranes comprise oceanic and continental crust or hydrated ultramafic material of density less than the upper mantle. Mafic eclogite mostly records peak conditions that correspond with the predicted density thresholds, which are in turn controlled by the subduction geotherm (60 and 80 km or *P* > 1.7 and 3.0 GPa; Fig. 1). Variability in the *P–T* conditions experienced by exposed mafic eclogite terranes has been attributed to the extent of mechanical coupling between the crust and the mantle wedge^35,36^. These are also influenced by the occurrence of serpentinite, which is buoyant (2.95–3.15 gcm^−3^) and prone to being intensely deformed during metamorphism^4,37–39^. The maximum depths from which mafic terranes may be exhumed are predicted to cluster at 30 and 80 km depending on interface-decoupling of the subduction channel from the overriding lithosphere, and at 55–60 km due to the effects of serpentinite dehydration (Fig. 1)^35,36^.

The bulk of the continental crust is predicted to remain positively buoyant at UHP conditions shallower than 120 km, beyond which its density progressively approaches 3.33 gcm^−3^ (*P* > 3.5 GPa: Fig. 2)^26^. Granitic crust is predicted to have a maximum burial depth of between 160 and 260 km (Figs. 3 and 4c)^19–21^, dependent on its water content. The presence of hydrous minerals (mafic or felsic) will affect rock density and prograde mineral progression, with dehydration reactions inducing large volume reductions at high-*PT* conditions following the consumption of water as a separate phase. Dry granitic crust will develop wadeite-bearing mineral assemblages and densities similar to that of the enclosing mantle at depths of 160–170 km (*P* = 5.5 GPa). The stabilisation of K-cymrite and phengite instead of wadeite in hydrous granitic crust is predicted to result in densities for such compositions remaining less than that of the ambient mantle to depths of 260 km, before marked densification associated with the growth of stishovite and K-hollandite (*P* = 8–10 GPa: Figs. 3b and 4c)^25^.

Mineral assemblages associated with UHP conditions general contain coesite, stishovite or majoritic garnet, and mostly occur in mafic or ultramafic protoliths hosted by comparatively buoyant felsic rock^26,40,41^. The co-occurrence of mafic or ultramafic rock with intermediate or felsic rock types is necessary to generate slab-pull forces that can drag the rocks to upper mantle conditions, before or after the detachment of any crust from its lithospheric mantle (Fig. 4). Such pull forces become increasingly important beyond burial to 80–90 km (2.5–2.8 GPa) due to a decrease in the rate of densification associated with thermal expansion (Fig. 4). For crust dominated by andesite to remain buoyant at UHP conditions (say 120 km or ~3.5 GPa), it need comprise no more than 25% mafic material (Fig. 4). These predictions can account for the occurrence of coesite, rare microdiamond and/or majoritic garnet from depths of up to 160 km in fragmented lithosphere (Figs. 2 and 5)^26,40^ that had limited basaltic material (<25%)^7–9^, as in several of the Himalayan UHP terranes^31^. Exhuming terranes with proportional more mafic or ultramafic material requires they be dominated by felsic material^6,26,42^. The subduction and exhumation of crust dominated by granite requires that there be less than 40% accompanying mafic component, for the crust to be positive buoyancy at depths between 160 and 260 km. Higher proportions of dense rock types will render mixed terranes susceptible to comparatively slow and/or staged exhumation^42^: they could, for example, initially lose a dense anchor at UHP conditions and be partial uplifted, to then have a second stage of mafic/ultramafic loss whilst stalled at the base (30–60 km or 0.5–1.8 GPa) of the overriding lithosphere (e.g. Dabie–Sulu, the Western Gneiss Terrane & Bohemian Massif^42–44^).

Comparatively few UHP occurrences are inferred to have returned from depths beyond 160 km (*P* > 5 GPa), but burial to depths of 360–400 km (*P* ≈ 12 GPa) have been attributed to rocks with pseudomorphous textures posited to be after stishovite, α-PbO_2_-type TiO_2_ and/or the presence of exsolution textures in majoritic garnet and omphacite^11–18^. The exhumation of eclogite from depths of 200–260 km is possible for granitic protoliths, particularly for those with hydrous high-grade assemblages (Fig. 4). However, the interpreted return of eclogite from depths of 300–400 km draws attention to the need of a plausible tectonic process (e.g. Altyn Tagh, Ezgebirge massif or Kokchetav massif)^12–14,17,45^. Mechanisms controlling the re-exposure of terranes with high proportions of mafic/ultramafic material from depths of 300–400 km is even more cryptic (e.g. Alpe Armi or Dabieshan)^11,16^.

## Returning From Ultra-Deep Burial

Caution seems warranted in attributing UHP assemblages to depths greater than 160 km (*P* > 5 GPa), in the absence of mechanisms that facilitated their positive buoyancy and uplift^2,6,9,26^. Alternative mechanisms that have been proposed as influential to the buoyant return of UHP terranes to Earth’s surface include their partial melting^45,46^ and/or the metastable persistence of lower-*P* phases^47^. Most UHP eclogite is hosted by comparatively buoyant felsic rock that is commonly retrogressed, and/or apparently does not record passage through UHP conditions, making it difficult to interpret their high-*P* history^26,40,41^. Relatively small disparities in burial *P* estimates (<0.5 GPa) can be attributed to tectonic overpressure, typically in localised high strain domains^41,48^.

At depths greater than 160 km, high ambient temperature conditions will reduce, but not eliminate the effects of kinetic impediments to metamorphic equilibration^49–51^. Generally, incomplete metamorphism is attributed to a lack of a free-fluid or hydroxyl-bearing phases that aid diffusion-controlled reaction kinetics^49–51^. Paradoxically, exhuming felsic crust from depths within the stishovite or wadeite eclogite facies (160–260 km) seems more feasible for compositions with hydrous mineral assemblages (Fig. 3). The inefficient metamorphism of dry granitic crust could result in either the complete metastable persistence of coesite and K-feldspar, or their partial transformation to wadeite or stishovite (<50% and <20%, respectively). Such inefficiency could result in crustal densities (3.33–3.43 gcm^−3^) close to the buoyancy limit at depths of 260 km. However, the catalysing influence of H_2_O makes reaction overstepping on depth-scales greater than 100 km seem less plausible for most subduction systems, particularly if hydrous and anhydrous rocks are intermingled^51^. The mineral assemblages observed in natural eclogite terranes reconcile well with predictions from fluid-saturated phase equilibria modelling, despite considerable variability in rock compositions and inferred equilibrium volumes (Table S2)^52,53^. It seems common that progressive metamorphic equilibration is achieved across a large range of *P–T* conditions^50,54^. It is also likely that fluid-saturated conditions were experienced by many exhumed UHP terranes, especially mafic compositions. Therefore, the subduction of wadeite or kyanite eclogite to depths of 350–400 km seems unlikely to escape localised metamorphic reaction and densification, and/or involve extensive metastable persistence^47^. Robust evidence for the subduction and exhumation of granitic crust from depths of 350–400 km might come by establishing pseudomorphs after K-cymrite, wadeite or K-hollandite as well as stishovite, and/or armoured inclusions and exsolution features that are linked to mineral exchange trends^13,14,21,55^.

## Methods

Phase equilibria modelling was performed using THERMOCALC^14^. Equilibria models for a MORB, serpentinite (Fig. S1) and an andesite were calculated in the NCKFMASHTO chemical system (Na_2_O–CaO–K_2_O–FeO–MgO–Al_2_O_3_–SiO_2_–H_2_O–TiO_2_–O) utilising version 3.45i^23^ and the internally consistent thermodynamic dataset 6.2 (updated 6^th^ February 2012)^28^. Lherzolite (Fig. S1) was modelled in the NCKFMASTOCr chemical system (Na_2_O–CaO–K_2_O–FeO–MgO–Al_2_O_3_–SiO_2_–TiO_2_–O–Cr_2_O_3_) for *P* = 1.0–3.5 GPa utilising version 3.47 of THERMOCALC and the internally consistent thermodynamic dataset 6.33 (updated 23^rd^ June 2017)^56^. Ultra-high-*P* conditions (4–15 GPa) in granite, MORB and lherzolite were modelled in the reduced NCKFMAS(H) (Na_2_O–CaO–K_2_O–FeO–MgO–Al_2_O_3_–SiO_2_–H_2_O) and NCFMAS(H) systems with the internally consistent dataset 6.2 (updated 6^th^ February 2012)^28,29^.

Mineral activity–composition models and abbreviations used for the NCKFMASHTO models include: glaucophane (gl), actinolite (act), hornblende (hb), omphacite/diopside (o/dio)^57^, feldspars (pl & kfs)^58^, garnet (g), paragonite (pa), biotite (bi), muscovite (mu), chlorite (chl)^59^, epidote (cz & zo), chloritoid (ctd), staurolite (st), talc (ta), olivine (ol)^28^, brucite (br) and antigorite (atg)^60^, with pure phases of lawsonite (law), albite (ab), rutile (ru), sphene (sph) quartz (q), coesite (coe), sillimanite/kyanite (sill/ky) and H_2_O. Mineral activity–composition models and abbreviations used for the NCKFMASTOCr system are: clinopyroxene (cpx), garnet (g), orthopyroxene (opx), spinel (sp), olivine (ol) and dry silicate liquid (liq)^56^. Mineral activity–composition models and abbreviations used in the NC(K)FMAS(H)^28,29^: orthopyroxene (opx), high-*P* clinoenstatite (hpx), clinopyroxene (o and jd), olivine (ol), wadsleyite (wad), majoritic garnet (g), corundum (cor), muscovite (mu), K-feldspar (ksp), together with pure phases coesite (coe), stishovite (stv), lawsonite (law), kyanite (ky), wadeite (wa) and K-hollandite (hol) and K-cymrite (kcm).

Pressure uncertainties for the assemblage field boundaries are approximately ±0.1 GPa at the 2σ level^61^, although the additional uncertainty on activity composition models will subtly increase this error. The densities of the modelled mineral assemblages were calculated within the THERMOCALC software, using the *calcsv* command^23^. Density estimates were corrected for free water using its defined Tait equation of state within the activity–composition relations of the thermodynamic dataset^28,62^. Non-linearity in the equation of state for H_2_O necessitated the calculation of its density variability at specific imposed conditions in relation to its modal proportions. The relative proportion of free water was determined by setting H_2_O at molar proportions that just saturated the low-temperature and high-pressure equilibria^38^ (Table S1). The equated density of all free water was subtracted from its thermodynamically equilibrated solid phase assemblage established in THERMOCALC. Uncertainty on the density calculations are likely to be <1% at the 2σ level^25,61^. Depth to pressure conversion was based on the PREM relationship^63^.

The modelled bulk rock compositions are based on a MORB-type eclogite from New Caledonia^38^, which shows limited effects of alteration across a large *PT* range^64^, an averaged andesitic composition of the continental crust^27^, an averaged of the upper continental crust of granitic composition^19^ and the KLB-1 lherzolite^65^. The modelled redox conditions for the MORB and the andesite were fixed at Fe^3+^/[Fe^3+^ + Fe^2+^] = 0.07–0.15, and the dry and hydrated lherzolite at 0.03, being considered appropriate for the crust and upper mantle, respectively^56,66^. Fluid was considered to be in excess in the MORB, serpentinite, andesite and granite models.

Two geothermal gradients for common subduction zones were used to interrogate the densities of oceanic and continental lithologies (Fig. S4). The cool geotherm is based on the Nankia subduction zone^67^, whereas the warm geotherm is based on the field array preserved on the Pam Peninsula, New Caledonia^38^, which approximates a geotherm that falls in between those interpreted for the modern Chilean and Cascadia subduction zones^68^. Both are consistent with exposed eclogite *P–T* estimates^35,69^.

Estimates of the pressure–temperature (*P–T*) conditions experienced by exposed high-pressure and UHP eclogite terranes worldwide were compiled and contrasted with conditions that could be predicted for their assemblages. The full compilation of eclogite terranes including mineral assemblages and published geothermobarometry results are shown in Table S2. As an additional comparison on the validity of *PT* and density estimates, the observed mineral assemblages were correlated with assemblages predicted by phase equilibria modelling (Table S2). Collectively, the compilation suggests that the phase equilibria calculations are generally robust for the gross considerations of the constituents of lithospheric buoyancy.

## Supplementary information

Supplementary methods

Supplementary table 2

## References

[1] Forsyth D, Uyeda S (1975). On the relative importance of the driving forces of plate motion. Geophys. J. Int..

[2] Ernst WG (2001). Subduction, ultrahigh-pressure metamorphism, and regurgitation of buoyant crustal slices – implications for arcs and continental growth. Earth Planet. Sci. Letts..

[3] Ringwood AE (1991). Phase transformation and their bearing on the constitution and dynamics of the mantle. Geoch. Et Cosm..

[4] Agard P, Yamato P, Jolivet L, Burov E (2009). Exhumation of oceanic blueschists and eclogites in subduction zones: timing and mechanisms. Earth-Sci. Rev..

[5] Fukao Y, Obayashi M, Inoue H, Nenbai M (1992). Subducting slabs stagnant in the mantle transition zone. J Geophys. Res..

[6] Hacker BR, Gerya TV (2013). Paradigms, new and old, for ultrahigh-pressure tectonism. Tectonophysics..

[7] Chopin C (1984). Coesite and pure pyrope in high-grade blueschists of the western Alps. A first record and some consequences. Contrib. Mineral. Petrol..

[8] Smith DC (1984). Coesite in clinopyroxene in the Caledonides and its implications for geodynamics. Nature..

[9] Sobolev NV, Shatsky VS (1990). Diamond inclusions in garnets from metamorphic rocks: a new environment for diamond formation. Nature..

[10] Van Roermund HIM, Drury MR (1998). Ultra-high pressure (*P* > 6 GPa) garnet peridotites in western Norway: exhumation of mantle rocks from >185 km depth. Terra Nova..

[11] Dobrzhinetskaya L, Green HW, Wang S (1996). Alpe Arami: a peridotite massif from depths of more than 300 kilometres. Science..

[12] Hwang S-L, Shen P, Chu H-T, Yui T-F (2000). Nanometer-size α-PbO_2_-type TiO_2_ in garnet: a thermobarometer for ultrahigh-pressure-metamorphism. Science.

[13] Liu L, Zhang J, Green HW, Jin Z, Bozhilov KN (2007). Evidence of former stishovite in metamorphosed sediments implying subduction to > 350 km. Earth Planet. Sci. Letts..

[14] Liu L (2018). Evidence of former stishovite in UHP eclogite from the South Altyn Tagh, western China. Earth Planet. Sci. Letts..

[15] Ye K, Cong B, Ye D (2000). The possible subduction of continental material to depths greater than 200 km. Nature.

[16] Song, S., Zhang, L., Chen, J., Liou, J. G. & Niu, Y. Sodic amphibole exsolutions in garnet from garnet-peridotite, North Qaidam UHPM belt, NW China: implications for ultradeep-origin and hydroxyl defects in mantle garnets. *Am*. *Mineral*., **90**, 814–820.

[17] Orgasawara Y, Fukasawa K, Maruyama S (2002). Coesite exsolution from supersilicic titanite in UHP marble from the Kokchetav Massif, northern Kazakhstan. Am. Mineral..

[18] Mposkos ED, Kostopoulos DK (2001). Diamond, former coesite and supersilicic garnet in metasedimentary rocks from the Greek Rhodope: a new ultrahigh-pressure metamorphic province established. Earth Planet. Sci. Letts..

[19] Irifune T, Ringwood AE, Hibberson WO (1994). Subduction of continental crust and terrigenous and pelagic sediments: an experimental study. Earth Planet. Sci. Letts..

[20] Wu Y, Fei Y, Jin Z, Liu X (2009). The fate of subducted upper continental crust: an experimental study. Earth Sci. Planet Letts..

[21] Zhang Y, Wu Y, Wang C, Zhu L, Jin Z (2016). Experimental constraints on the fate of subducted upper continental crust beyond the “depth of no return”. Geoch. Cosmo. Act.

[22] Chopin C (2003). Ultrahigh-pressure metamorphism: tracing continental crust into the mante. Earth Planet. Sci. Letts..

[23] Powell R, Holland TJB (1988). An internally consistent dataset with uncertainties and correlations: 3. Applications to geobarometry, worked examples and a computer program. J. Metamorph. Geol..

[24] Chen Y, Ye K, Wu TF, Guo S (2011). Exhumation of oceanic eclogites: thermodynamic constraints on pressure, temperature, bulk composition and density. J. Metamorph. Geol..

[25] Chapman, T., Clarke, G. L., Piazolo, S., & Daczko, N. R. Evaluating the importance of metamorphism in the foundering of continental crust. *Scientific Reports*. **7**, 10.1038/s41598-017-13221-6 (2017). 10.1038/s41598-017-13221-6 PMC5638824 29026119

[26] Massonne H-J, Willner AP, Gerya T (2007). densities of metapelitic rocks at high to ultrahigh pressure: what are the geodynamic consequences? *Earth Planet Sci*. Letts..

[27] Rudnick, R. L. & Gao, S. Composition of the continental crust. In: L Rudnick, R. L. (Ed.) *Treatise on Geochemistry*, 3. Elsevier-Pergamon, pp. 1–64 (2003).

[28] Holland TJB, Powell R (2011). An improved and extended internally consistent thermodynamic dataset for phases of petrological interest, involving a new equation of state for solids. J. Metamorph. Geol..

[29] Holland TJB, Hudson NFC, Powell R, Harte B (2013). New thermodynamic models and calculated phase equilibria in NCFMAS for basic and ultrabasic compositions through the transition zone into the uppermost lower mantle. J. Petrol..

[30] Mackenzie D, Jackson J, Priestley K (2005). Thermal structure of oceanic and continental lithosphere. Earth Planet. Sci. Letts..

[31] Weller OM, Copley A, Miller WGR, Palin RM, Dyck B (2019). The relationship between mantle potential temperature and oceanic lithosphere buoyancy. Earth Planet. Sci. Letts..

[32] Chemenda AI, Mattauer M, Malavielle J, Bokun AN (1995). A mechanism for syn-collisional rock exhumation and associated normal faulting: results from physical modelling. Earth Planet. Sci. Letts..

[33] Davies JH, von Blanckenburg F (1995). Slab breakoff: a model of lithosphere detachment and its test in the magmatism and deformation of collisional orogens. Earth Planet. Sci. Letts..

[34] Gerya T, Yuen DA, Maresch WV (2004). Thermomechanical modelling of slab detachment. Earth Planet. Sci. Letts..

[35] Agard P, Plunder A, Angiboust S, Bonnet G, Ruh J (2018). The subduction plate interface: rock record and mechanical coupling (from long to short timescales). Lithos.

[36] Whitney Donna L., Teyssier Christian, Seaton Nicholas C. A., Fornash Katherine F. (2014). Petrofabrics of high-pressure rocks exhumed at the slab-mantle interface from the “point of no return” in a subduction zone (Sivrihisar, Turkey). Tectonics.

[37] Hernández-Uribe, D. & Palin, R. M. A revised petrological model for subducted oceanic crust: Insights from phase equilibrium modelling. *J*. *Metamorph*. *Geol*., 10.1111/jmg12483.

[38] Clarke GL, Powell R, Fitzherbert JA (2006). The lawsonite paradox: a comparison of field evidence and mineral equilibria modelling. J. Metamorph. Geol..

[39] Angiboust S, Agard P (2010). Initial water budget: the key to detaching large volumes of eclogitized oceanic crust along the subduction channel?. Lithos..

[40] Gilotti J (2013). The realm of ultrahigh-pressure metamorphism. Elements..

[41] Palin RM, Reuber GS, White RW, Kaus BJP, Weller OM (2017). Subduction metamorphism in the Himalayan ultrahigh-pressure Tso Morari massif: an integrated geodynamic and petrological modelling approach. Earth Planet. Sci. Letts..

[42] Kylander-Clark ARC, Hacker BR, Mattinson CG (2012). Size and exhumation rate of ultrahigh-pressure terranes linked to orogenic stage. Earth Planet. Sci. Letts..

[43] Walsh EO, Hacker BR (2004). The fate of subducted continental marins: two-stage exhumation of the high-pressure to ultrahigh-pressure Western Gneiss Region, Norway. J. Metamorph. Geol..

[44] Medris LG, Beard BI, Jelínek E (2004). mantle-derived, UHP garnet pyroxenite and eclogite in the Moldanubian Gföhl Nappe, Bohemian Massif: a geochemical review, New P–T determinations, and tectonic interpretation. Int. Geol. Rev..

[45] Dong, J., Wei, C.-J., Clarke, G. L. & Zhang, J.-X. Metamorphic evolution during deep subduction and exhumation of continental crust: insights from felsic granulites in South Altyn Tugh, West China. *J*. *Petrol*., 10.1093/petrology/egy086/5102820 (2018).

[46] Baldwin SL (2004). Pliocene eclogite exhumation at plate tectonic rates in eastern Papua New Guinea. Nature..

[47] Peterman EM, hacker BR, Baxter EF (2009). Phase transformations of continental crust during subduction and exhumation: Western Gneiss Region, Norway. Eur. J. Mineral..

[48] Reuber, G., Kaus, B. J. P., Schmalholz, S. M. & White, R. W. Nonlithostatic pressure during subduction and collision and the formation of (ultra)high-pressure rocks. *Geology*, **44**, 343–346.

[49] Powell R, Guiraud M, White RW (2005). Truth and beauty in metamorphic phase-equilibria: conjugate variables and phase diagrams. Can. Mineral..

[50] Chapman T, Clarke GL, Piazolo S, Daczko NR (2019). Inefficient high-temperature metamorphism in orthogneiss. Am. Mineral..

[51] Chapman Timothy, Clarke Geoffrey L., Piazolo Sandra, Robbins Victoria A., Trimby Patrick W. (2019). Grain‐scale dependency of metamorphic reaction on crystal plastic strain. Journal of Metamorphic Geology.

[52] Wei CJ, Clarke GL (2011). Calculated phase equilibria for MORB compositions: a reappraisal of the metamorphic evolution of lawsonite eclogite. J. Metamorph. Geol..

[53] Marmo BA, Clarke GL, Powell R (2002). Fractionation of bulk rock composition due to porphyroblast growth: effects on eclogite facies mineral equilibria, Pam Peninsula, New Caledonia. J. Metamorph. Geol..

[54] Powell R., Evans K. A., Green E. C. R., White R. W. (2018). On equilibrium in non-hydrostatic metamorphic systems. Journal of Metamorphic Geology.

[55] Langenhorst F, Poirer J-P (2009). ‘Eclogitic’ minerals in shocked basaltic meteorite. Earth Planet. Sci. Letts..

[56] Holland TJB, Green ECR, Powell R (2018). melting of peridotites through to granites: a simple thermodynamic model in the system KNCFMASHTOCr. J. Petrol..

[57] Green ECR (2016). Activity–composition relations for the calculations of partial melting equilibria for metabasic rocks. J. Metamorph. Geol..

[58] Holland TJB, Powell R (2003). Activity–composition relations for phases in petrological calculations: an asymmetric multicomponent formulation. Cont. Mineral. Petrol.

[59] White RW, Powell R, Holland TJB, Johnson TE, Green ECR (2014). New mineral activity–composition relations for thermodynamic calculations in metapelitic systems. J. Metamorph. Geol..

[60] Evans KA, Powell R (2015). The effect of subduction on the sulphur, carbon and redox budget of lithospheric mantle. J. Metamorph. Geol..

[61] Powell R, Holland TJB (2008). On thermobarometry. J. Metamorph. Geol..

[62] Pitzer KS, Sterner SM (1995). Equations of state valid continuously from zero to extreme pressures with H_2_O and CO_2_ as examples. Int. J. Thermodyn..

[63] Dziewonski AM, Anderson DL (1981). Preliminary reference Earth model. Phys. Earth Planet. Interiors..

[64] Spandler C, Hermann J, Arculus RJ, Mavrogenes J (2003). Redistribution of trace elements during prograde metamorphism from lawsonite blueschist to eclogite facies: implications for deep subduction-zone processes. Contrib. Mineral. Petrol..

[65] Davies FA, Tangeman JA, Tenner TJ, Hirschmann MH (2009). The composition of KLB-1 peridotite. Am. Mineral..

[66] Kelley KA, Cottrell E (2009). Water and oxidation state of subduction zone magmas. Science.

[67] Peacock SM, Wang K (1999). Seismic consequences of warm versus cool subduction metamorphism: examples from Southwest and Northeast Japan. Science..

[68] Syracuse EM, van Keken PE, Abers GA (2010). The global range of subduction zone thermal models. Phys. Earth Planet. Interiors..

[69] Penniston-Dorland SC, Kohn MJ, manning CE (2015). The global range of subduction zone thermal structures from exhumed blueschists and eclogites: rocks are hotter than models. Earth Planet. Sci. Letts..

