# Supplementary material for: The role of buoyancy in the fate of ultra-high-pressure eclogite
Source: Sci Rep. 2019 Dec 27;9:19925. doi: 10.1038/s41598-019-56475-y (PMC6934836; doi:10.1038/s41598-019-56475-y)
Supplement: Supplementary file 1 — Supplementary methods [file 41598_2019_56475_MOESM1_ESM.pdf]

Supplement: The role of buoyancy in the fate of ultra-high-pressure eclogite

Timothy Chapman[1]*^, Geoffrey L. Clarke[1] & Nathan R. Daczko[2]

[1]*School of Geosciences, The University of Sydney, NSW, 2006, Australia*
[2]ARC Centre of Excellence for Core to Crust Fluid Systems and GEMOC, Department of Earth and Planetary Sciences, Macquarie University, NSW, 2109, Australia
*Corresponding author: t.chapman@sydney.edu.au
^current address: *Earth Science, School of Environmental and Rural Science, University of New England, NSW, 2351, Australia*

Phase equilibria and densities

The simple lithospheric column utilised in the modelling does not account for natural compositional heterogeneity in both the crust and lithospheric mantle. Therefore, the phase equilibria results present a minimum density estimate. However, much of the uncertainty is negated by the use of average compositions, and the fact that heterogeneity is typically restricted to small portions of the lithosphere (Table S1). To completely assess variability in the density relations of the continental crust an additional model of a fluid-saturated metasedimentary protolith was considered in the NCKFMASHTO system (14NC15G: Fig. S4). The density relations are generally similar though slightly less than the bulk andesitic crustal composition (~0.05 gcm^{-3}: Figs S3 & S6). This dictates a greater proportion of a mafic anchor to enable the obtainment of UHP conditions. Changing the proportion of crust or mantle (1–5 km) influences the densities calculations by <6%.

As most rock types returning from UHP conditions are supracrustal in origin, fluid saturated conditions are considered the most appropriate, and correlate best to exposed natural mineral assemblages (*see Natural Observations*). Fluid was considered to be in excess in the serpentinite (Fig. S1), MORB (Fig. S2), andesite (Fig. S3), and granite models, consistent with observations in natural high-pressure terranes (Schmidt & Poli, 1998). At UHP conditions many rock-types are comparatively dry and assessed here using both dry and wet granite combined with MORB in reduce systems (Fig. S5). In turn, much of the mafic lower continental crust is relatively dry and less dense than a fluid-saturated MORB composition, but still denser than 3.33 gcm^{-3} at P > 1.7 GPa and T > 700˚C (Chapman et al., 2017). To reduce inherit biases and uncertainties the models do not explicitly consider metastable persistence (Powell et al., 2019).

The phase equilibria and densities of dry granite at upper mantle conditions correlate to the experiment relations on the same composition by Irifune et al. (1994) and Zhang et al. (2016). Even subtle variations in bulk compositions influence the modal proportions of jadeite and garnet (Wu et al., 2009). Although these differences do not impact the predicted depth-limits in absolute terms. The transformation of kyanite to corundum at P = 12–14 GPa is distinct from experimental relations involving the stabilisation of aluminium-silicate phase (CAS = $CaAl_4Si_2O_{11}$: Irifune et

al., 1994) on account of the lack of a suitable end-member in the current dataset. However, kyanite stability is consistent with decomposition relations from experimentation and thus considered reasonable in terms of high-pressure phase equilibria (Schmidt et al., 1997). The wet granite phase equilibria are consistent with results in simpler chemical systems, predicting the stability of phengite and K-cymrite instead of wadeite (Fasshauer et a., 1997; Schmidt et al., 2004). In both dry and wet systems, the solidi were not calculated due to insufficient model constraints for a haplogranitic liquid at UHP conditions (Holland & Powell, 2011). Experimental results predict the dry and wet solidi at *T* between 1100–1600˚C, above the modelled geothermal gradients utilised in this work (Irifune et al., 1994).

The geotherms represent conditions that intersect peak conditions typically associated with exhumed eclogite terranes worldwide. Natural *P–T* estimates are considered more appropriate to assess subduction geotherms as model based thermal regimes partially underestimate *T* (Penniston-Dorland et al., 2015). The geotherms do not consider cold slab interiors, that would have the effect of extending the depth limits. As most of the material returned from high-*P* conditions are restricted to the upper crust, it is the slab surface temperatures that are more appropriate for determining depths of no return. The oceanic lithosphere densities show broad similarity to those of Weller et al. (2019), which explicitly assess thermal variability within the subducting plate.

Natural observations

A full compilation of eclogite terranes including mineral assemblages and published geothermobarometry results are shown in Supplementary Table S2. This compilation is distinct from those of Agard et al. (2018) and Brown & Johnson (2019) as it considers uncertainty in *P–T* estimates and the recorded mineral assemblages.

The diversity in bulk-composition for felsic UHP assemblages makes any direct comparison to the phase equilibria predictions based on an andesitic, granitic and metasedimentary bulk composition problematic. Traditional geothermobarometry for eclogite facies rocks, such as Fe-Mg exchange in garnet and clinopyroxene is compounded by large uncertainties, the effects of which can be largely reduced utilising the pseudosection approach (e.g. Štipská & Powell, 2005; Powell & Holland, 2008). Together with variability in the extent of equilibration associated with sluggish reaction kinetics during subduction metamorphism, the incomplete transformation of the lithosphere during subduction is commonly invoked (e.g. Peterman et al., 2007). However, there is a strong correlation between the observed *versus* posited mineral assemblages in mafic and ultramafic rock types at peak conditions. In these instances, phase equilibria predicted for the *PT* conditions inferred from geothermobarometry match the natural mineral assemblages with a tolerance of one or two phases.

The correlation is consistent with: (1) equilibrium having been attained on a reasonable scale, though some differences can be accounted for by domainal equilibration phenonium, such as elemental fractionation (e.g. Marmo et al., 2002;

Chapman et al., 2019a) or variability in bulk composition (e.g. Wei & Clarke, 2011; Chapman et al., 2017); (2) the metastable persistence of minerals, at least locally, appears restricted in mafic compositions (e.g. Chapman et al., 2019b); (3) water-saturated conditions are appropriate across most of the *PT* conditions, as nearly all assemblages have the predicted stable hydrous minerals (see also Schmidt & Poli, 1998); (4) the modelled densities are accurate within these uncertainty bounds; and (5) estimates on the *PT* conditions are appropriate within the extent of the modelled phase equilibria fields and the uncertainty on these calculations. The main caveat is these results do not account for sampling biases in the original publications that can exclude rocks which depart from equilibrium.

Table S1 Whole-rock compositions used for phase equilibria modelling shown in weight and mole percent oxide.

	MORB	andesite	lherzolite	serpentinite	granite	Metased.
	14NC14	bulk-continent	KLB-1	KLB-1		14NC15G
Wt.%	Clarke et al. (2006)	Rudnick & Gao (2003)	Davies et al. (2009)		Irifune et al. (1994)	Clarke et al. (2006)
SiO_2	48.03	60.60	44.48	44.48	66.20	57.75
TiO_2	1.68	0.72	0.11	0.11	0.60	1.82
Al_2O_3	14.96	15.90	3.51	3.51	15.90	16.98
FeO	10.60	6.04	8.20	8.20	4.60	8.28
MnO	0.15	0.10	0.12	0.12	-	0.19
MgO	7.44	4.66	39.52	39.52	1.90	3.62
CaO	12.38	6.41	3.07	3.07	4.80	2.10
Na_2O	1.97	3.07	0.30	0.30	2.70	2.55
K_2O	0.14	1.81	0.02	0.02	3.40	3.02
Cr_2O_3	-	-	0.32	0.32	-	-
SUM	97.35	99.31	99.65	99.65	100.10	99.61
mol. %						
H_2O	excess	excess	-	excess	-/excess	excess
SiO_2	50.84	64.36	38.27	38.27	71.82	63.89
Al_2O_3	9.33	9.95	1.78	1.78	10.16	11.07
CaO	14.05	7.29	2.83	2.83	5.58	2.49
MgO	11.75	7.38	50.68	50.68	3.07	5.97
FeO	9.38	5.36	5.90	5.90	4.17	7.66
K_2O	0.09	1.23	0.01	0.01	2.35	2.14
Na_2O	2.02	3.16	0.25	0.25	2.84	2.74
TiO_2	1.33	0.58	0.07	0.07	-	1.52
O	1.20	0.69	0.10	0.10	-	0.39
Cr_2O_3	-	-	0.11	-	-	

Supplementary References

Agard, P., Plunder, A., Angiboust, S., Bonnet, G., Ruh, J. The subduction plate interface: rock record and mechanical coupling (from long to short timescales). *Lithos*, **320–321**, 537–566. (2018).

Brown, M., Johnson, T. Metamorphism and the evolution of subduction on Earth. *Am. Mineral.*, **104**, 1065–1082. (2019).

Chapman, T., Clarke, G.L., Piazolo, S., & Daczko, N.R. Evaluating the importance of metamorphism in the foundering of continental crust. *Scientific Reports* **7**, DOI:10.1038/s41598-017-13221-6. (2017).

Chapman, T., Clarke, G. L., Piazolo, S., Robbins, V. A. & Trimby, P. Grain-scale dependency of metamorphic reaction of crystal plastic strain. *J. Metamorph. Geol*. DOI: 10.1111/jmg.12473, (2019).

Chapman, T., Clarke, G. L., Piazolo, S. & Daczko, N. R. Inefficient high-temperature metamorphism in orthogneiss. *Am. Mineral.* **104**, 17–30, (2019).

Clarke, G. L., Powell, R. & Fitzherbert, J. A. The lawsonite paradox: a comparison of field evidence and mineral equilibria modelling. *J. Metamorph. Geol.* **24**, 715–725. (2006).

Davies, F. A., Tangeman, J. A., Tenner, T. J. & Hirschmann, M. H. The composition of KLB-1 peridotite. *Am. Mineral.* **94**, 176–180. (2009).

Fasshauer, D. W., Chatterjee, N. D., Marler, B. Synthesis, structure, thermodynamic properties, and stability relations of K-cymrite, K[$AlSi_3O_8$]-H_2O. Phys. Chem. Minerals. **24**, 455–462. (1997).

Holland, T. J. B. & Powell, R. An improved and extended internally consistent thermodynamic dataset for phases of petrological interest, involving a new equation of state for solids. *J. Metamorph. Geol.* **29**, 333–383 (2011).

Irifune, T., Ringwood, A. E., Hibberson, W. O. Subduction of continental crust and terrigenous and pelagic sediments: an experimental study. *Earth Planet. Sci. Letts.*, **126**, 351–368. (1994).

Marmo, B. A., Clarke, G. L. & Powell, R. Fractionation of bulk rock composition due to porphyroblast growth: effects on eclogite facies mineral equilibria, Pam Peninsula, New Caledonia. *J. Metamorph. Geol.* **20**, 151–165. (2002).

Penniston-Dorland, S. C., Kohn, M. J., manning, C. E. The global range of subduction zone thermal structures from exhumed blueschists and eclogites: rocks are hotter than models. *Earth Planet. Sci. Letts.*, **428**, 243–254. (2015).

Powell, R., Holland, T. J. B. On thermobarometry. *J. Metamorph. Geol.* **26**, 155–179 (2008).

Rudnick, R. L., Gao, S. Composition of the continental crust. In: L Rudnick, R. L. (Ed.) *Treatise on Geochemistry*, 3. Elsevier-Pergamon, pp. 1–64 (2003).

Schmidt, M. W., Poli, S., Comodi, P., Zanazzi, P. F. High-pressure behavior of kyanite: decomposition of kyanite into stishovite and corundum. *Am. Mineral.*, **82**, 460–466. (1997).

Schmidt, M. W. & Poli, S. Experimental based water budgets for dehydrating slabs and consequences for arc magma generation. *Earth. Planet. Sci. Letts.* **163**, 361–379. (1998).

Schmidt, M. W., Vielzeuf, D., Auzanneau, E. Melting and dissolution of subducting crust at high pressures: the key role of white mica. *Earth Planet. Sci. Letts.*, **228**, 65–84. (2004).

Štípská, P. & Powell, R. Constraining the *P–T* path of a MORB-type eclogite using pseudosections, garnet zoning and garnet–clinopyroxene thermometry: an example form the Bohemian Massif. *J. Metamorph. Geol.* **23**, 725–743 (2005).

Syracuse, E. M., van Keken, P. E. & Abers, G. A. The global range of subduction zone thermal models. *Phys. Earth Planet. Interiors.* **183**, 73–90. (2010).

Weller, O. M., Copley, A., Miller, W. G. R., Palin, R. M., Dyck, B. The relationship between mantle potential temperature and oceanic lithosphere buoyancy. *Earth Planet. Sci. Letts.*, **518**, 86–99. (2019).

Wei, C. J. & Clarke, G. L. Calculated phase equilibria for MORB compositions: a reappraisal of the metamorphic evolution of lawsonite eclogite. *J. Metamorph. Geol.* **29**, 939–952. (2011).

Wu, Y., Fei, Y., Jin, Z., Liu, Z. The fate of subducted upper continental crust: an experimental study. *Earth Planet. Sci. Letts.*, **282**, 275–284. (2009).

Zhang, Y., Wu, Y., Wang, C., Zhu, L., Jin, Z. Experimental constraints on the fate of subducted upper continental crust beyond the "depth of no return". *Geoch. Cosmo. Act*, **186**, 207–225. (2016).

Exposed eclogite references

Altherr, R., Topuz, G., Marschall, H., Zazk, T. & Lugwig, T. Evolution of a tourmaline-bearing lawsonite eclogite from the Elekdag area (Central Pontides, N Turkey): evidence for infiltration of slab-derived B-rich fluids during exhumation. *Contrib. Mineral. Petrol.* **148,** 409–425. (2004).

Angiboust, S. & Agard, P. Initial water budget: the key to detaching large volumes of eclogitized oceanic crust along the subduction channel? *Lithos*. **120**, 453–474. (2010).

Angiboust, S., Langdon, R., Agard, P., Waters, D. & Chopin, C. Eclogitization of Monviso ophiolite (W. Alps) and implications on subduction dynamics. *J. Metamorph. Geol.* **30**, 3–61. (2012).

Baldwin, S. L., Monteleone, B. D., Webb, L. E., Fitzgerald, P. G., Grove, M. & Hill, J. Pliocene eclogite exhumation at plate tectonic rates in eastern Papua New Guinea. *Nature*. **431**, 263–267. (2004).

Bozhilov, K. N., Green, H. W., Dobrzhinetskaya, L. Clinoenstatite in Alp Arami peridotite: additional evidence of very high pressure. *Science*, **284**, 128–132. (1999).

Caby, R. Precambrian coesite from Northern Mali: first record and implications for plate tectonics in the trans-Saharan segment of the Pan-African belt. *Eur. J. Mineral.* **6**, 235–244. (1994).

Cao, Y., Song, S. G., hiu, Y. L., Jung, H. & Jin, Z. M. Variation of mineral composition, fabric and oxygen fugacity from massive to foliated eclogites during exhumation of subducted ocean crust in the North Qilian suture zone, NW China. *J. Metamorph. Geol.* **29,** 699–720. (2011).

Chopin, C. Coesite and pure pyrope in high-grade blueschists of the western Alps. A first record and some consequences. *Contrib. Mineral. Petrol.* **86**, 107–118. (1984).

Clarke, G. L., Aitchison, J. C. & Cluzel, D. Eclogites and blueschists of the Pam Peninsula, NE New Caledonia: a reappraisal. *J. Petrol.* **38,** 843–876. (1997).

Compagnoni, R. & Rolfo, F. UHPM units in the western Alps. E*MU notes Mineral.* **5**, 13–49. (2003).

Davis, P. B. & Whitney, D. L. Petrogenesis of lawsonite and epidote eclogite and blueschist, Sivrihisar Massif, Turkey. *J. Metamorph. Geol.* **24**, 823–849. (2006).

Davoudian, A. V., Genser, J., Dachs, E. & Shabanian, N. petrology of eclogites from north of Shahrekord, Sanandaj-Sirjan zone, Iran. *Mineral. Petrol.* **92,** 393–413. (2008).

DesOrmeau, J. W., Gordon, S. M., Little, T. A., Bowring, S. A. & Chatterjee, N. Rapid time scale of Earth's youngest known ultrahigh-pressure metamorphic event, Papua New Guinea. *Geology*, **45**, 795–798. (2017).

Dobrzhinetskaya, L., Green, H. W. & Wang, S. Alpe Arami: a peridotite massif from depths of more than 300 kilometres. *Scienc*e. **271**, 1841–1845. (1996).

Dong, J., Wei, C-J., Clarke, G. L. & Zhang, J-X. Metamorphic evolution during deep subduction and exhumation of continental crust: insights from felsic granulites in South Altyn Tugh, West China. *J. Petrol.* DOI: 10.1093/petrology/egy086/5102820. (2018)

Fitzherbert, J. A., Clarke, G. L. & Powell, R. Lawsonite–omphacite-bearing metabasites of the Pam Peninsula, NE New Caledonia: evidence for disrupted blueschist- to eclogite-facies conditions. *J. Petrol.* **44,** 1805–1831. (2003).

Fitzherbert, J. A., Clarke, G. L. & Powell. R. Preferential retrogression of high-*P* metasediments and the preservation of blueschist to eclogite facies metabasite during exhumation, Diahot terrane, NE New Caledonia. *Lithos*. **83,** 67–96. (2005).

Gardien, V., Tegyey, M., Lardeaux, J. M., Misseri, M. & Dufour, E. Crust–mantle relationships in the French Variscan chain: the example of the Southern Monts du Lyonnais unit (eastern French Massif central. *J. Metamorph. Geol.* **8**, 477–492. (1990).

Gilotti, J. A. & Ravna, E. J. K. first evidence for ultrahigh-pressure metamorphism in the North-East Greenland Caledonides. *Geology*. **30,** 551–554. (2002).

Groppo, C., Beltrando, M. & Compagnoni, R. The *P–T* path of ultra-high pressure Lago Di Cignana and adjoining high-pressure meta-ophiolitic units: insights into the evolution of the subducting Tethyan slab. *J. Metamorph. Geol.* **27**, 207–231. (2009).

Ghent, E., Tinkham, D. & Marr, R. Lawsonite eclogites from the Pinchi Lake area, British Columbia – new *P–T* estimates and interpretation. *Lithos*. **109,** 248–253. (2009).

Glassley W. E., Kaorstgård, J. A., Sørensen, K., Platou, S. W. A new UHP metamorphic complex in the ~1.8 Ga Nagssugtoqidian Orogen of West Greenland. *Am. Mineral.*, **99**, 1315–1334. (2014).

Hirajima, T., Banno, S., Hiroi, Y. & Ohta, Y. Phase petrology of eclogites and related rocks from the Motalafjella high-pressure metamorphic complex in Spitsbergen (Arctic Ocean) and its significance. *Lithos*. **22,** 75–97. (1988).

Hwang, S., Shen, P., Chu, H., Yui, T. nanometer-size α-PbO_2-type TiO_2 in garnet: a thermobarometer for ultrahigh-pressure metamorphism. *Science*, **288**, 321–325. (2000).

Janák, M., Froitzheim, N., Georgiev, N., Nagel, T. J., Sarov, S. *P–T* evolution of kyanite eclogite from the Pirin Mountains (SW Bulgaria): implications for the

Rhodope UHP metamorphic complex. *J. metamorph. Geol.*, **29**, 317–332. (2011).

John, T., Scherer, E. E., Schenk, V., Herms, P., Halama, R. & Garbe-Schönberg, D. Subducted seamounts in an eclogite-facies ophiolite sequence: he Andean Raspas Complex, SW Ecuador. *Contrib. Mineral. Petrol.* **159,** 265–284. (2010).

Lardeaux, J. M., Ledru, P., Daniel, I & Duchene, S. The Variscan French Massif central – a new addition to the ultrahigh-pressure metamorphic 'club': exhumation processes and geodynamic consequences. *Tectonophysics*. **332**, 143–167. (2001).

Laurent, V., Lanari, P., Näir, I., Augier, R., Lahfid, A. & Jolivet, L. Exhumation of eclogite and blueschist (Cyclades, Greece): pressure–temperature evolution determined by thermobarometry and garnet equilibrium modelling. *J. Metamorph. Geol.* **36,** 769–798. (2017).

Liou, J. G., Zhang, R. Y. & Jahn, B. Petrological and geochemical characteristics of ultrahigh-pressure metamorphic rocks from the Dabie-Sulu terrane, East-Central China. *Int. Geol Rev*. **42**, 328–352. (2000).

Liu, D., Jian, P., Kröner, A. & Xu, S. dating of prograde metamorphic events deciphered from episodic zircon growth in rocks of the Dabie-Sulu UHP complex, China. *Earth Planet. Sci. Letts.* **250**, 650–666. (2006).

Liu, X., Jin, Z., Green, H. W. Clinoenstatite exsolution in diopsidic augite of Dabieshan: Garnet peridotite from depth of 300 km. *Am. Mineral.*, **92**, 546–552. (2007).

Liu, L., Wang, C., Cao, Y-T., Chen, D-L., Kang, L., Yang, W-Q. & Zhu, X-H. Geochronology of multi-stage metamorphic events: constraints on episodic zircon growth from the UHP eclogite in the South Altyn, NW China. *Lithos*. **139–139,** 10–26. (2012).

Liu, L., Zhang, J., Cao, Y., Green, H. W., yang, W., Xu, H., Liao, X., Kang, L. Evidence of former stishovite in UHP eclogite from the South Altyn Tagh, western China. *Earth Planet. Sci. Letts.*, **484**, 353–362. (2018).

Lü, Z., Zhang, L., Du, J. & Bucher, K. Petrology of coesite-bearing eclogite from Habutengsu Valley, western Tianshan, NW China and its tectonometamorphic implication. *J. Metamorph. Geol.* **27,** 773–787. (2009).

Massone, H. A comparison of the evolution of diamondiferous quartz-rich rocks from the Saxonian Erzgebirge and the Kokchetav Massif: are so-called diamondiferous gneisses magmatic rocks? *Earth Planet. Sci. Letts.*, **216**, 347–364. (2003).

Medaris, L. G., Beard, B. & Jelínek, E. Mantle-derived, UHP garnet pyroxenite and eclogite I the Moldanubian Gföhl Nappe, Bohemian Massif: a geochemical review, new *P–T* determinations, and tectonic interpretation. *Int. Geol. Rev.* **48,** 765–777. (2004).

Miyazaki, K., Zulkarnain, I., Sophaheluwakan, J. & Wakita, K. Pressure–temperature conditions and retrograde paths of eclogites, garnet–glaucophane rocks and

schists from South Sulawesi, Indonesia. *J. Metamorph. Geol.* **14,** 549–563. (1996).

Mposkos, E. D., Kostopoulos, D. K. Diamond, former coesite and supersilicic garnet in metasedimentary rocks from the Greek Rhodope: a new ultrahigh-pressure metamorphic province established. *Earth Planet. Sci. Letts.*, **192**, 497–506. (2001).

Nimis, P. & Morten, L. *P–T* evolution of 'crustal' garnet peridotites and included pyroxenites from Nonsberg area (upper Austroalpine), NE Italy: from the wedge to the slab. *J. Geodynm.* **30**, 93–115. (2000).

Nimis, R. & Trommsdorf, V. Revised thermobarometry of Alpe Arami and other garnet peridotites from the central Alps. *J. Petrol.* **42**, 103–115. (2001).

O'Brien, P. J., Zotov, N., Law, R., Khan, M. A. & Jan, M. Q. Coesite in Himalayan eclogite and implications for models of India-Asia collision. *Geology*. **29**, 435–438. (2001).

Och, D. J., Leitch, E. C., Caprarelli, G. & Watanabe, T. Blueschist and eclogite in tectonic melange, Port Macquarie, New South Wales, Australia. *Mineral. Mag.* **67,** 609–624. (2003).

Orgasawara, Y., Fukasawa, K., Maruyama, S. Coesite exsolution from supersilicic titanite in UHP marble from the Kokchetav Massif, northern Kazakhstan. *Am. Mineral.*, **87**, 454–461. (2002).

Palmeri, R., Ghiribelli, B., Ranalli, G., Talarico, F. & Ricci, C. A. Ultrahigh-pressure metamorphism and exhumation of garnet-bearing ultramafic rocks from the Lanterman Range (northern Victoria Land, Antartica). *J. Metamorph. Geol.* **25**, 225–243. (2007).

Palin, R. M., Reuber, G. S., White, R. W., Kaus, B. J. P. & Wellerm O. M. Subduction metamorphism in the Himalayan ultrahigh-pressure Tso Morari massif: an integrated geodynamic and petrological modelling approach. *Earth Planet. Sci. Letts.* **467**, 108–119. (2016).

Rad, G. R. F., Droop, G. T. R., Amini, S. & Moazzen, M. Eclogites and blueschists of the Sistan suture zone, eastern Iran: a comparison of *P–T* histories from a subduction mélange. *Lithos*. **84**, 1–24. (2005).

Ravna, E. J. K. & Terry, M. P. Geothermobarometry of UHP and HP eclogites and schists – an evaluation of equilibria among garnet–clinopyroxene–kyanite–phengite–coesite/quartz. *J. Metamorph. Geol.* **22**, 579–592. (2004).

Schneider, J., Bosch, D., Monié, P., Guillot, S., García-Casco, A., Lardeaux, J. M., Torres-Roldán, R. & Trujillo, G. M. Origin and evolution of the Escambray Massif (Central Cuba): an example of HP/LT rocks exhumed during intraoceanic subduction. *J. Metamorph. Geol.* **22,** 227–247. (2002).

Shibakusa, H. & Mawkawa, H. Lawsonite-bearing eclogitic metabasites in the Cazadero area, northern California. *Mineral. Petrol.* **61**, 163–180. (1997).

Sobolev, N. V., Shatsky, V. S. Diamond inclusions in garnets from metamorphic rocks: a new environment for diamond formation. *Nature*, **343**, 742–746. (1990).

Song, S., Zhang, L., Chen, J., Liou, J. G., Niu, Y. Sodic amphibole exsolutions in garnet from garnet-peridotite, North Qaidam UHPM belt, NW China: implications for ultradeep-origin and hydroxyl defects in mantle garnets. *Am. Mineral.*, **90**, 814–820.

Spengler, D., van Roermund, H. L. M., Drury, M. R., Ottolini, L., Mason, P. R. D., Davies, G. R. Deep origin and hot melting of an Archaean orogenic peridotite massif in Norway. *Nature*, **440**, 913–917. (2006).

Terry, M. P., Robinson, P. & Ravna, E. J. K. Kyanite eclogite thermobarometry and evidence for thrusting of UHP over HP metamorphic rocks, Nordøyane, Western Gneiss region, Norway. *Am. Mineral.* **85**, 1637–1650. (2000).

Tsujimori, T., Sisson, V. B., Liou, J. G., Harlow, G. & Sorensen, S. S. Very-low-temperature record of subduction process: a review of worldwide lawsonite eclogite. *Lithos*. **92,** 609–624. (2006).

Usui, T., Nakamurra, E., Kobayashi, K., Maruyana, S. & Helmstaedt, H. Fate of the subducted Farallon plate inferred from eclogite xenoliths in the Colorado Plateau. *Geology*. **31,** 589–592. (2003).

Van Roermund, H. L. M., Drury, M. R., Barnhoorn, A. & De Ronde, A. Relict majoritic garnet microstructures from ultra-deep orogenic peridotites in Western Norway. *J. Petrol*. **42**, 117–130. (2001).

Vignaroli, G., Rossetti, F., Bouybaouene, M., Massonne, H-J., Theyem T, Faccenna, C. & Runiciello, R. A counter-clockwise *P–T* path for the Voltri Massif eclogites (Liguruan Alps, Italy). *J. Metamorph. Geol.* **23,** 533–555. (2005).

Vitale Brovarone, A. V., Groppo, C., Hetényi, G, Compagnoni, R. & Malavielle, J. Coexistence of lawsonite-bearing eclogite and blueschist: phase equilibria of Alpine Corsica metabasalts and petrological evolution of subducting slabs. *J. Metamorph. Geol.* **29,** 583–600. (2011).

Wallis, S. & Aoya, M. A re-evaluation of eclogite facies metamorphism in SW Japan: proposal for an eclogite nappe. *J. Metamorph. Geol.* **18**, 653–664. (2000).

Whitney, D. L. & Davies, P. B. Why is lawsonite eclogite so rare? Metamorphism and preservation of lawsonite eclogite, Sivrihiar, Turkey. *Geology* **34**. 473–476. (2006).

Ye, K., Cong, B., Ye, D. The possible subduction of continental material to depths greater than 200 km. *Nature*, **407**, 734–738. (2000).

Zack, T., Rivers, T., Brumm, R. & Kronz, A. Cold subduction of oceanic crust: implication from a lawsonite eclogite from the Dominican Republic. *Eur. J. Mineral.* **16**, 909–916. (2004).

Zhang, L., Ellis, D. J., Arculus, R. J., Jiang, W. & Wei, C. 'Forbidden zone' subduction of sediments to 150 km depth – the reaction of dolomite to magnesite + aragonite in the UHPM metapelites from western Tianshan, China. *J. Metamorph. Geol.* **21**, 523–529. (2003).

Zhang, J. X., Mattinson, C. G., Meng, F. C. & Wan, Y. S. An Early Palaeozoic HP/HT granulite–garnet peridotite association in the South Altyn Tagh, NW

China: *P–T* history and U–Pb geochronology. *J. Metamorph. Geol.* **23**, 491–510. (2005).

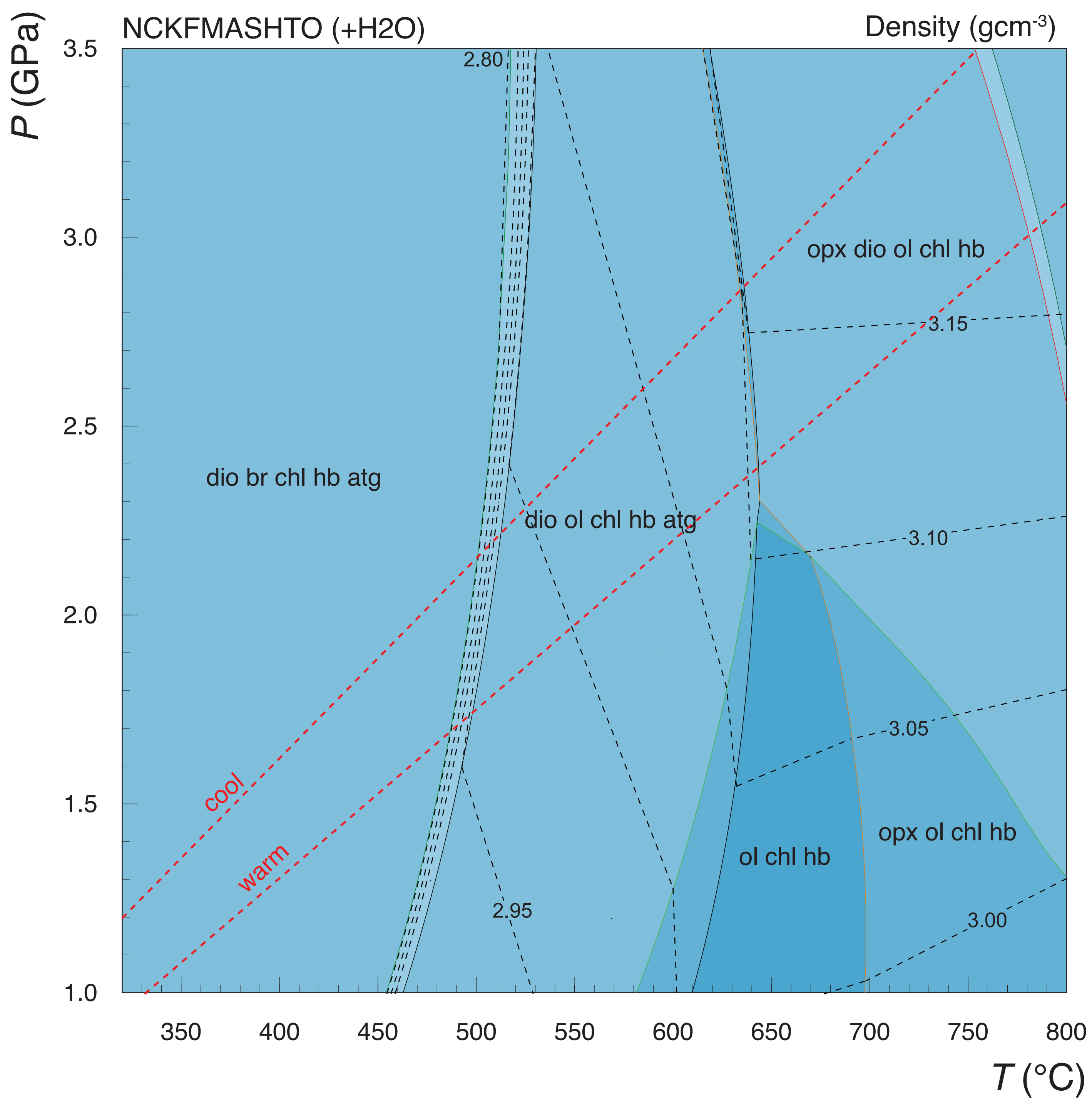

Figure S1 *P–T* pseudosection for a wet lherzolite. Different shading of the fields represents changes in variance. Isopycnals are indicated in dashed black lines.

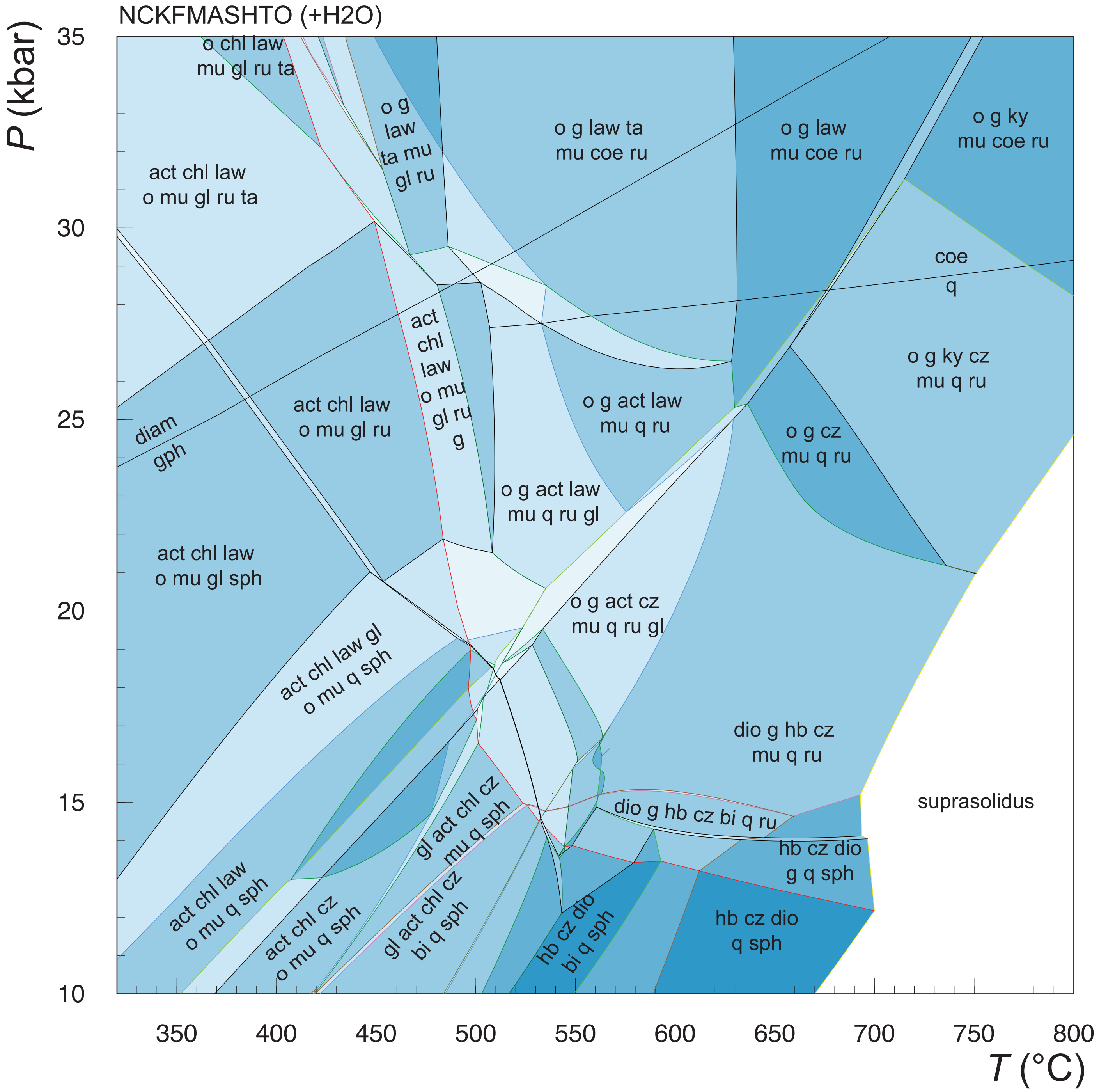

Figure S2 *P*–*T* pseudosection calculated for a MORB bulk-rock composition (Table S1). The phase assemblage fields correspond to those shown in Figure 1 of the main text.

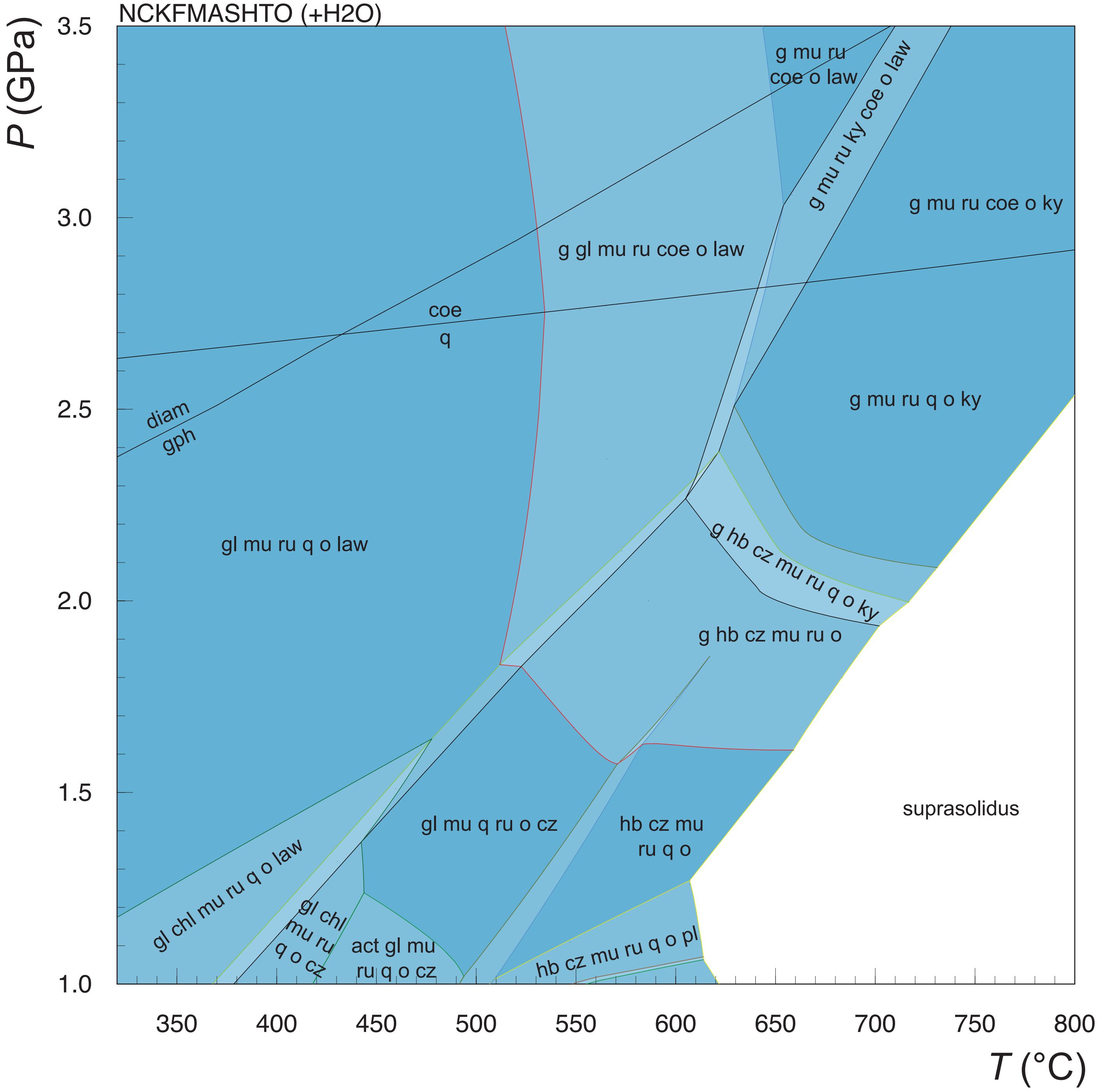

Figure S3 *P*–*T* pseudosection calculated for an andesitic composition that is considered an averaged composition of the continental crust (Table S1). The phase assemblage fields correspond to those shown in Figure 2 of the main text.

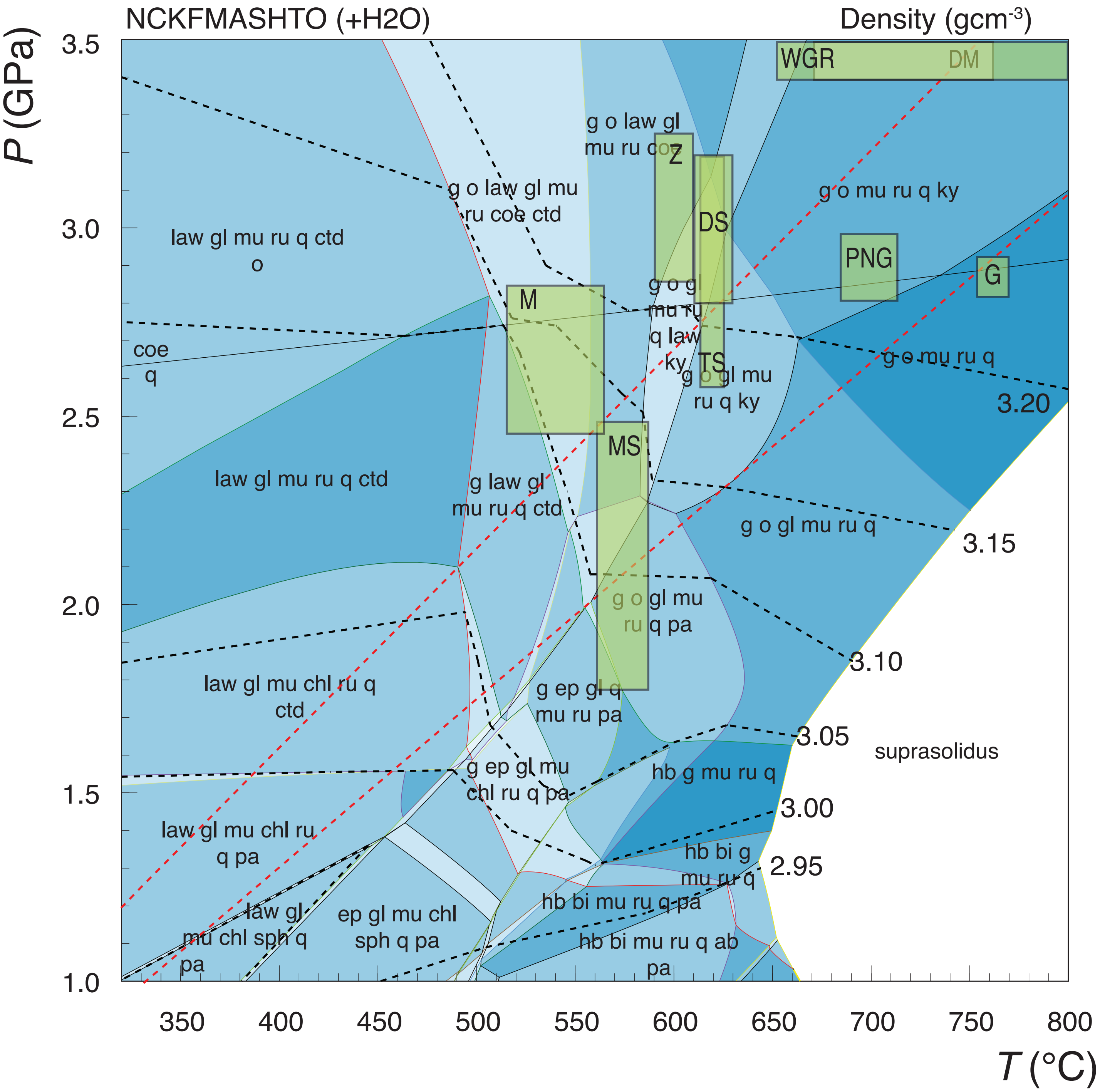

Figure S4 *P–T* pseudosection for a metapelite. Different shading of the fields represents changes in variance. Isopycnals are indicated in dashed black lines with the labelled boxes indicating the *P–T* conditions inferred for assemblages in natural UHP eclogite terranes.

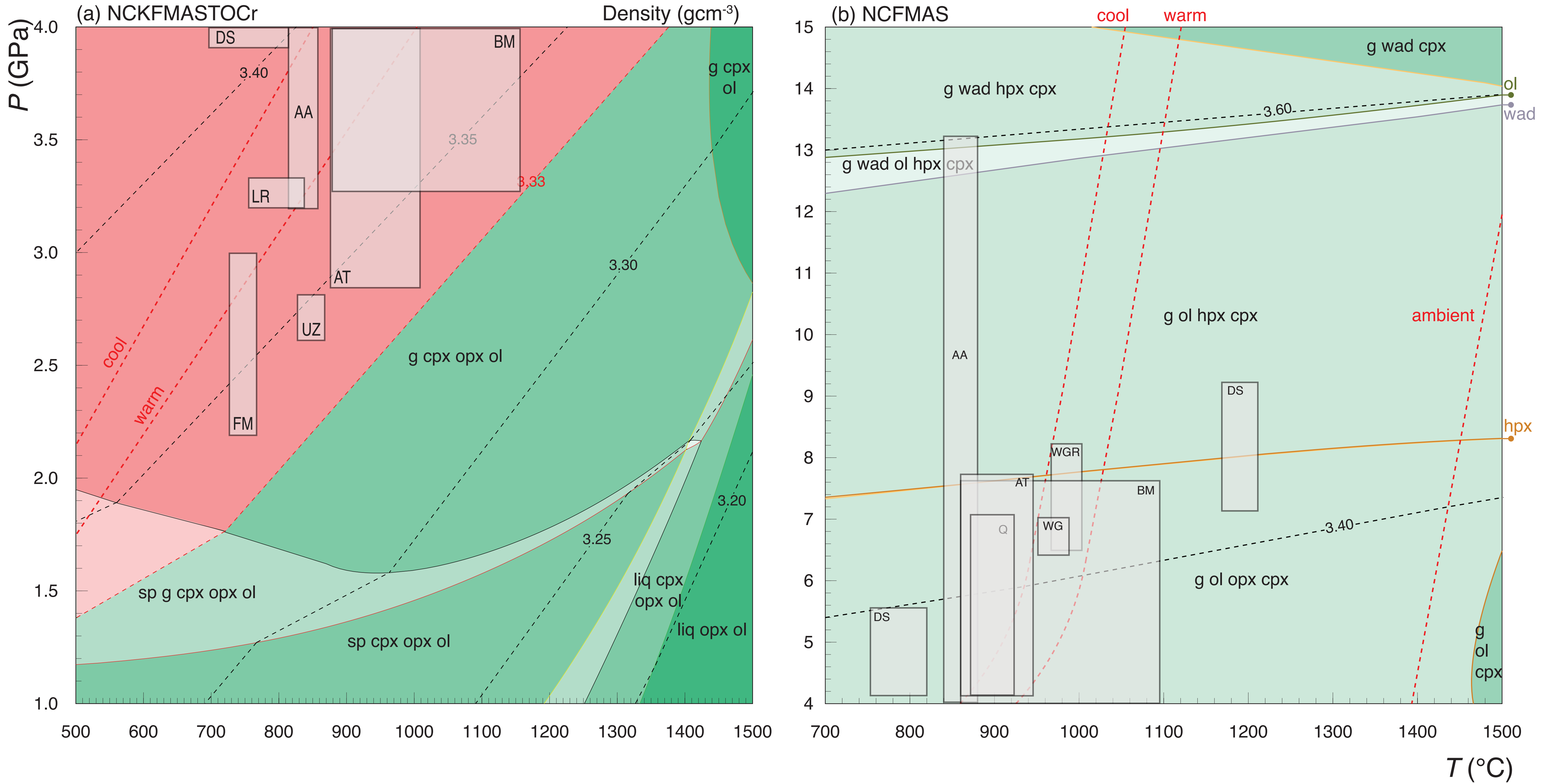

Figure S5 *P–T* pseudosections for lherzolite at low-presure in the (a) NCKFMASTOCr system and high-pressure in the (b) NCFMAS system. Different shading of the fields represents changes in variance, with the red fields in (a) indicating assemblages with a density greater than 3.3 g cm^{-3}. Isopycnals are indicated in dashed black lines with the labelled boxes indicating the *P–T* conditions inferred for assemblages in natural UHP terranes.

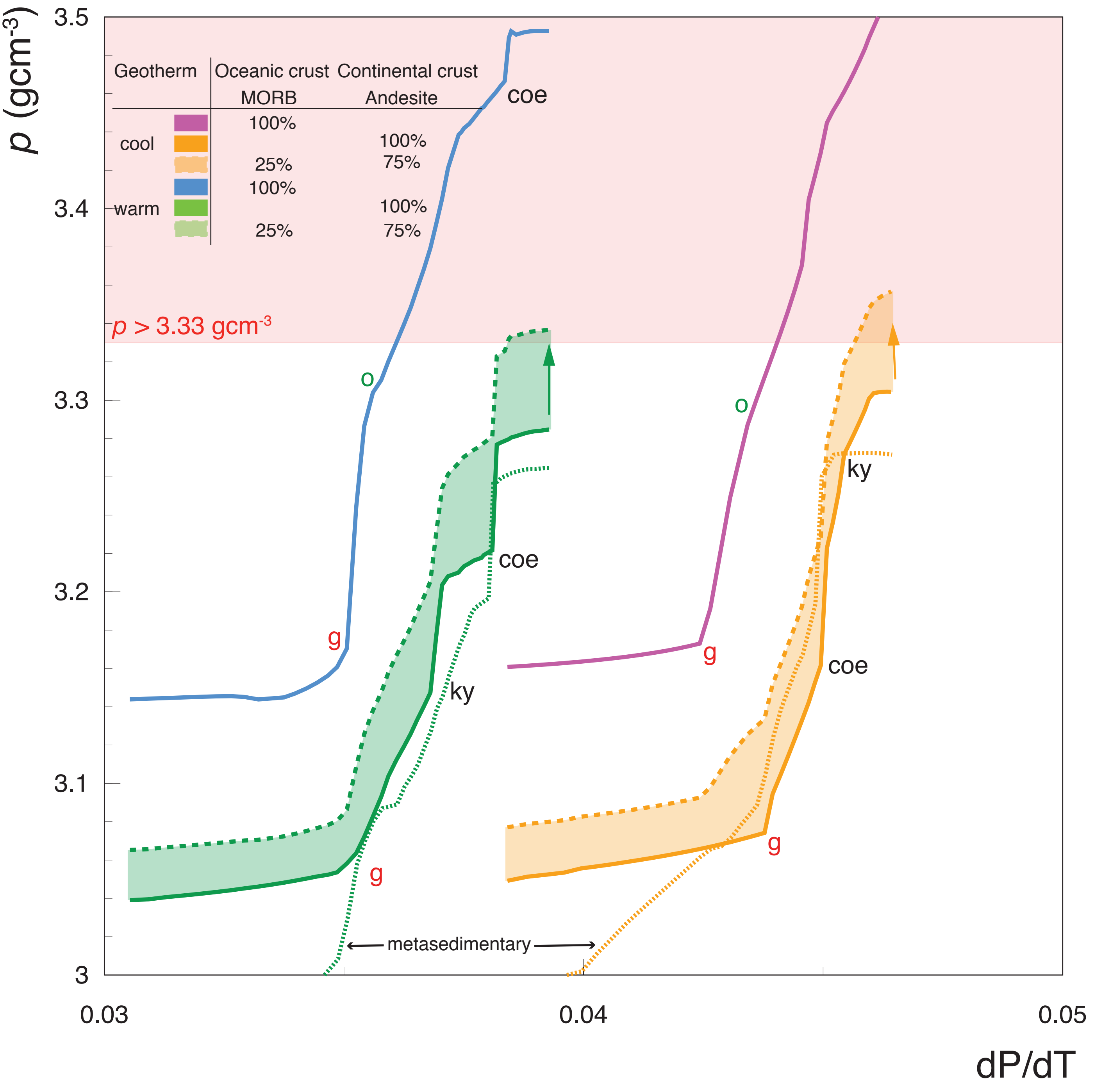

Figure S6 Density changes predicted for the mineral equilibria in oceanic and continental crust undergoing cool and warm subduction (reflected in the *x*-axis, *dP/dT*). The diagram displays the key density thresholds for the two conditions of subduction utilising appropriate conjugate variables, and providing the complement to Figuew 3 in the Main Text. Depth calculations are derivative of the *P–T* estimates, and density is an extensive variable with a conjugate pairing to the intensive pressure variable.
